# WHO multimodal strategy for improving hand hygiene supported within the organization’s management system, Mater Dei Hospital, Belo Horizonte, Brazil

**DOI:** 10.1186/2047-2994-4-S1-P152

**Published:** 2015-06-16

**Authors:** SB Ricardo, LC Belo, PT Leite, AM Lara, RF Rocha

**Affiliations:** 1 Infection Control Department, Hospital Mater Dei, Belo Horizonte, Brazil

## Introduction

Hand hygiene (HH) has been singled out as a core element of patient safety for the prevention of healthcare-associated infections (HAIs) and the spread of antimicrobial resistance. Cleansing hands with alcohol-based hand rub is a simple and undemanding procedure that requires only a few seconds. Although we already had the five components of the WHO Multimodal Hand Hygiene Improvement Strategy implemented in our facility and a steady mandatory training program, compliance remains stable, with no further increase for many months.

## Objectives

The objective of this study was to improve compliance of HH among HCWs by enhancing the monitoring and feedback, periodical analysis by leaders and with stronger support from the hospital administration.

## Methods

On admission, patients were asked to monitor HCW adherence to HH and fulfill the customer satisfaction survey at the time of hospital discharge. The results were converted into indicators, with monthly feedback of the infection control team for leaders. It was set at 77% in 2012, 80% in 2013 and 83% in 2014. The results became part of the Profit Distribution Program for employees, already well-established in the organization. The results were validated by the sample direct observation by trained nurses and by comparison with alcohol consumption rates per 1,000 patient-days.

## Results

In January 2012 the general adherence was 75% and reached 83% in December 2014 (an average increase of 10%). The adherence of nurses and physicians ranged from 74% to 84% and 73% to 82%, respectively. There was no significant difference between hospital wards. During this period the average annual rate of alcohol consumption for hand rub was 18.2, 24.8 and 44.4 liters per 1000 patient-days. During this 3 years the HAIs rates remained low (<2%).

## Conclusion

Ownership for compliance must come from within clinical teams, and not solely driven from the infection control team. Support from the hospital management is essential. All tools available in the institution should be identified and can be used to promote patient safety climate. The participation of patients, continuous feedback and the economic stimulus were successful to help motivate HCW to improve HH.

## Disclosure of interest

None declared.

